# Genome sequence of bovine herpesvirus 5 (BoHV-5) isolate from India

**DOI:** 10.1128/mra.00950-24

**Published:** 2024-12-31

**Authors:** Assim Verma, Himanshu Kamboj, Nitin Khandelwal, Ram Kumar, Thachamvally Riyesh, Naveen Kumar

**Affiliations:** 1 National Centre for Veterinary Type Cultures, ICAR-National Research Centre on Equines, Hisar, India; Katholieke Universiteit Leuven, Leuven, Belgium

**Keywords:** bovine herpesvirus 5, *Herpesviridae*, meningoencephalitis, transboundary disease

## Abstract

Recently, we identified bovine herpesvirus 5 (BoHV-5) in a vaginal swab from aborted cattle. It was unusual in two aspects: first, its association with abortion (it is otherwise mainly associated with encephalitis), and second, it is the first report from India (as it is mostly restricted to South American countries). In this study, we conducted the genome sequencing of the BoHV-5 isolate and provided insights into its phylogenetic relationships with other BoHV-5 strains. These findings contribute valuable knowledge toward understanding the epidemiology and evolution of BoHV-5.

## ANNOUNCEMENT

Bovine herpesvirus 5 (BoHV-5), a neurotropic virus from the *Varicellovirus* genus in the *Herpesviridae* family, causes severe meningoencephalitis in cattle (1). BoHV-5 is genetically and antigenically close to BoHV-1 (2), which primarily affects the respiratory and reproductive systems of infected cattle.

Although BoHV-5 cases are sporadic in some other countries (3), it poses significant challenges in South America, especially in Brazil and Argentina (4, 5). Our group recently reported BoHV-5 infection in Indian cattle (2). Initially, based on its isolation from an aborted cattle, cross-neutralization with BoHV-1, and amplification of BoHV-1 gene segments in PCR, it was identified to be BoHV-1 (2). However, later, *UL27, UL44,* and *UL54* gene-based nucleotide sequencing and differential (BoHV-1/BoHV-5) PCR, confirmed it to be BoHV-5 (2). Unlike typical encephalitic cases, this BoHV-5 strain was isolated from the vaginal swab of an aborted cow (2).

With limited whole-genome data on BoHV-5 (only seven complete genomes from South America are available; Table 1), understanding its evolution and global epidemiology remains challenging. In this report, we present the genome sequence of the BoHV-5 isolate from India.

**TABLE 1:** List of Bovine alpha herpesvirus 5 complete genome sequences available on the NCBI portal

Sr. No.	Accession number	Sequence length	Origin	Collection year	Strain
1	PP897810.1	138,358	India	2018	2018/Bhilwara
2	MZ420492.1	138,185	Argentina	2010	674/10
3	NC005261.3	138,390	Brazil	1999	SV507/99
4	KY549446.1	137,712	Brazil	1997	ISO 97/45
5	KY559403.2	137,741	Brazil	1996	P160/96
6	MZ364295.1	137,976	Argentina	1984	166/84
7	MW829288	138,036	Argentina	1982	A663

For virus isolation, vaginal swab from recently aborted cattle was collected in a viral transport medium, filtered through a 0.22 µm syringe filter, and mixed (100 µL filtrate) with 400 µL of antibiotic-supplemented Dulbecco’s Modified Eagle’s Medium (DMEM) to infect Madin-Darby Bovine Kidney cells for 2 h, followed by replacement with fresh DMEM and 7 days of incubation at 37°C in a CO_2_ incubator. Cytopathic effects were observed during the third blind passage. For sequencing, the infected culture supernatant was concentrated by ultracentrifugation, and DNA was extracted (QIAGEN DNA kit).

Nucleotide sequencing was performed on the Illumina NovaSeq 6000 system using QIseq Fx DNA library preparation protocol. Quality checks and adapter trimming were conducted using FastQC v0.11.8 and Trim Galore 0.6.6. A total of 13,209,222 paired-end reads (151 bp) were mapped to the Bovine herpesvirus type 1.1 genome (isolate NVSL challenge 97–11; accession no. JX898220.1) using BWA mem 0.7.17, with mapped reads extracted via Samtools v1.11. *De novo* assembly using Unicycler v0.4.9 (SPAdes 3.15.5) yielded a high-quality genome (138,358 bp, 74.8% GC, 28,832.34× coverage). The genome was annotated using the VGAS tool (http://cefg.uestc.cn/vgas).

To contextualize Indian BoHV-5 isolate globally, we conducted a phylogenetic analysis. Multiple sequence alignment performed using the MAFFT webserver (https://mafft.cbrc.jp/alignment/server/index.html) with available complete BoHV-5 genome sequences suggested that cattle (semen) trade may have introduced BoHV-5 into India.

**Fig 1:**
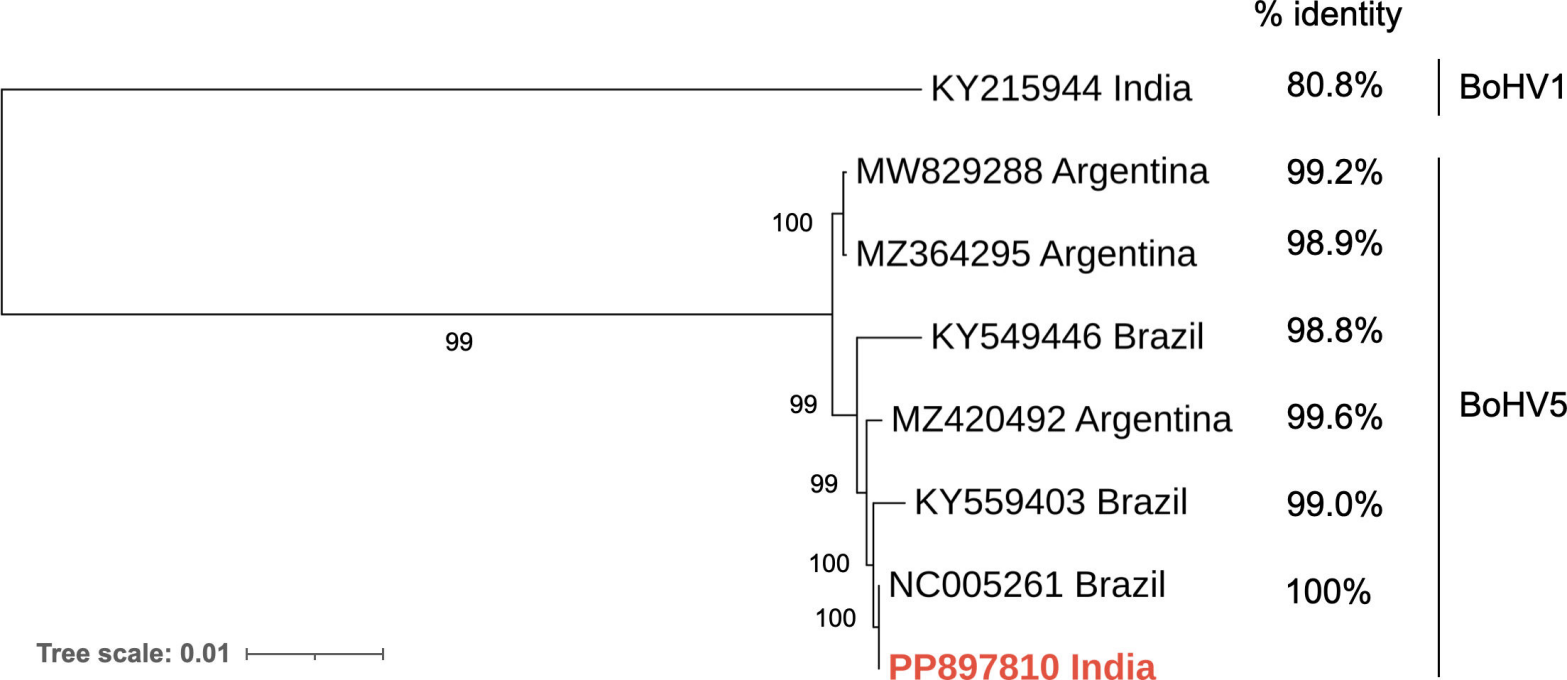
Phylogenetic tree and respective whole genome-based nucleotide percent identity. Method: neighbor-joining, model: Jukes-Cantor (1,000 bootstrap replicates). Indian BoHV-1 genome as outgroup. The tree was visualized using ITOL (https://itol.embl.de/tree).

The analysis revealed a closer genetic relationship between the Indian BoHV-5 isolate and Brazilian strains compared with Argentinian isolates (Fig. 1), suggesting that cattle (semen) trade may have introduced BoHV-5 into India.

The coding-complete genome sequence of this Indian BoHV-5 strain provides a critical addition to the limited genomic data on this pathogen. It offers key insights into the molecular epidemiology of BoHV-5 in India and underscores the need for ongoing surveillance and research to understand its transmission dynamics and impact on cattle health in the region.

## Data Availability

This Whole Genome sequence has been deposited in NCBI portal (https://www.ncbi.nlm.nih.gov/genbank/) under the accession no. PP897810.1. The raw reads were deposited at the NCBI Sequence Read Archive (SRA) under the accession number SRR30427815.
